# Psychometrics of inflammatory back pain criteria in the US population

**DOI:** 10.1016/j.ero.2025.04.006

**Published:** 2025-05-16

**Authors:** Mark C. Hwang, Seokhun Kim, Shervin Assassi, Alexis Ogdie, Michael H. Weisman, John Reveille, Charles Green

**Affiliations:** 1 Division of Rheumatology, Department of Internal Medicine, John P. and Kathrine G. McGovern School of Medicine at The University of Texas Health Science Center at Houston, Houston, TX, USA; 2 Center for Clinical Research & Evidence-Based Medicine, Department of Pediatrics, John P. and Kathrine G. McGovern School of Medicine at The University of Texas Health Science Center at Houston, Houston, TX, USA; 3 Division of Rheumatology, Department of Medicine, Perelman School of Medicine, University of Pennsylvania, Philadelphia, PA, USA; 4 Division of Immunology/Rheumatology, Department of Medicine, Stanford University School of Medicine, Palo Alto, CA, USA

## Abstract

**Objectives:**

Evaluation of axial spondyloarthritis relies on capturing various inflammatory back pain (IBP) features represented in different criteria. These criteria are likely to be interpreted differently across demographic subpopulations. We examined IBP criteria dimensionality and differential item functioning (DIF) among different age, gender, and race groups using a nationally representative sample.

**Methods:**

We utilised the National Health and Nutrition Examination Survey (NHANES) 2009–2010 dataset, a US nationally representative data collection with demographic weighting. The Arthritis Questionnaire (ARQ) within NHANES included questions from published Calin, European Spondyloarthropathy Study Group, and Berlin criteria (8a and 7b). Confirmatory factor analysis (CFA) was performed to assess the dimensionality of IBP criteria. DIF analysis based on Item Response Theory was applied to evaluate differences in criterion performance across age (20–49 years vs >50 years), gender (men vs women), and race (white vs non-white) groups.

**Results:**

The study included 1511 patients with complete ARQ IBP questionnaires. CFA indicated that 1-factor models were generally preferred for all IBP criteria. DIF analysis revealed age-related differences in several IBP items, with non-uniform DIF observed except in the Berlin 8a criteria. In contrast, gender differences were noted primarily in the Berlin 8a lower back pain item, and racial differences were minimal.

**Conclusions:**

Findings support the predominant use of a single underlying construct in IBP criteria, validating their clinical application. However, demographic factors, especially age and gender, introduce significant variability in responses, necessitating tailored approaches in clinical assessment and further research to confirm these findings across broader populations.

## INTRODUCTION

Inflammatory back pain (IBP) is a hallmark symptom of axial spondyloarthritis (axSpA), and its assessment is essential in the diagnosis, management, and classification of this condition. Established criteria aim to capture the characteristic features of IBP and include the Calin, European Spondyloarthropathy Study Group (ESSG), Berlin, and Assessment of SpondyloArthritis international Society (ASAS) criteria [1–4]. The criteria development has employed various methods—single-centre expert opinion, comparisons of radiographic axSpA (r-axSpA) patients to chronic lower back pain patients, and a study of suspected spondyloarthritis (SpA) patients by a group of international SpA experts, all with a focus on classification rather than diagnosis. Furthermore, IBP criteria have been used successfully as a SpA screening parameter by primary care physicians and orthopedists [5].

Studies examining the prevalence of IBP are scarce and vary widely in their estimates. The estimated age-adjusted IBP prevalence is 5.0%-6.0% in the general US population (US National Health and Nutrition Examination Survey [NHANES]) depending on the applied IBP criteria [6]. Prevalence did not appear to differ based on gender, but differences occurred across race and ethnicities. Other community-based studies worldwide have also studied IBP prevalence based on different IBP criteria and methodologies. A large, community practice-based study in Norfolk, UK, estimated the range of IBP prevalence as 1.7%-3.4% [7]. An epidemiologic study in Mexico City found IBP prevalence of 1.3% based on the Berlin IBP criteria [8]. A study of a low-income, low-health literacy population from Brazil estimated IBP prevalence of 14.9%-28.3%, depending on the IBP criteria [9]. Among university employees, a study in Turkey applying the different IBP criteria estimated prevalence of 6.6%-21.5% [10]. A study of university students estimated the prevalence of IBP to be 2.9% in southern China using the ASAS IBP criteria [11].

These large variations in IBP prevalence, as well as the varying degree of relationship to concomitant SpA prevalence in these respective countries, create questions regarding the application and interpretability of IBP criteria positivity at a population level and clinical setting [12]. We hypothesised that IBP exists at a population level as a continuous, latent condition/ailment. We also hypothesised that there is differential measurement performance for IBP criteria, or IBP may conceptually be different in specific subpopulations based on age, gender, and ethnicity. We sought to elucidate the different IBP criteria interpretability by studying their dimensionality and performance across diverse subpopulations from a nationally representative cohort.

## METHODS

### Study population and dataset

The study analysed data from the NHANES 2009–2010 dataset. NHANES is a cross-sectional, nationally representative survey that monitors the health status of civilian, non-institutionalised US population. The participants were selected through a multistage, probability design. Oversampling major US demographic subgroups, including Hispanics, non-Hispanic blacks, and low-income populations. In NHANES 2009–2010, a total of 6684 persons aged 20 to 59 years were screened, and 5103 completed records for a participation rate of 76.4%. We previously reported the demographic distribution of NHANES 2009–2010 by age, gender, race and ethnicity, and back pain characteristics, as summarised in Table 1 [6].

The Arthritis Questionnaire (ARQ) was designed for and used in NHANES 2009–2010 to estimate the prevalence of IBP and r-axSpA (historically referred to as ankylosing spondylitis during the time of ARQ development) in the US population, with questions from Calin, ESSG, and Berlin 7b and 8a IBP criteria [13–15]. The ARQ created text-based questions for IBP parameters, including morning stiffness, pain awakening from sleep and pain response to exercise, all formulated at an eighth-grade reading level. These questions were derived from a previous US study’s ankylosing spondylitis (AS) case ascertainment tool and further cognitively tested, revised, and piloted in 2008 [16]. Previous publications describe the details regarding the ARQ questionnaire development and IBP parameters [6,13].

### Statistical analysis

Descriptive statistics of the NHANES respondents and the study sample were calculated. All missing values were imputed using the missForest method, which has been shown in simulation studies to outperform other techniques, such as multivariate imputation by chained equations [17]. We also did complete case-only analyses only for sensitivity analyses.

### Dimensionality analysis

To assess whether IBP criteria effectively measure a single or multiple underlying concepts, we used confirmatory factor analysis (CFA), a statistical method that tests whether different symptoms cluster together as part of the same condition. This allowed us to determine whether IBP-related symptoms clustered together as a single condition (latent IBP) or needed to be considered as separate entities (eg, IBP and nonspecific back pain). A good model fit indicated that IBP criteria effectively captured a singular underlying construct. Model fit was assessed using chi-square difference testing, where a significant result favoured a 2-factor model for better fit, whereas a nonsignificant result supported the 1-factor model for parsimony. Reported model fit indices included the comparative fit index and root mean square error of approximation (RMSEA).

All IBP items were binary categorical variables, and model estimation was performed using mean- and variance-adjusted weighted least squares, applying a probit function for each factor loading to maintain proper model identification.

### Differential item functioning analysis

After establishing the dimensionality of the IBP criteria sets, we applied differential item functioning (DIF) analysis, a technique that detects bias in questionnaire items based on patient characteristics to evaluate whether IBP criteria perform differently across demographic groups. The grouping variables for DIF were age (20–49 years vs >50 years), gender (men vs women), and race (white vs non-white), respectively. To examine age-related differences, we applied both a dichotomisation at 50 years and a median-based stratification to explore potential cutoff variability. This approach ensures a robust analysis of IBP criteria across different age groups, particularly older patients who may experience different symptom presentations.

DIF analysis using Item Response Theory (IRT) with 2-parameter logistic model evaluated whether the items function differently for one subgroup as compared to the other with respect to the latent IBP scale after controlling for the overall differences in the constructs between subgroups. With an anchor item selected from the constrained baseline approach [18], chi-squared-based model comparison procedure was performed to identify DIF for each item with p value threshold being adjusted with Bonferroni method (0.05 divided by the number of tested items). If the chi-square difference was significant for an item, DIF testing for slope and intercept, performed separately, determined whether the item was more discriminative for one subgroup than the other (slope difference; non-uniform DIF) and/or the probability of item response was uniformly different for the 2 subgroups (intercept or difficulty difference; uniform DIF). Item difficulty refers to the point on the latent IBP scale where the population has 50% probability of responding ‘yes’ to a particular IBP item. The uniform DIF in the context of this study indicates that the item is consistently harder to say ‘yes’ for one subgroup with a higher difficulty estimate across the entire range of the latent IBP scale than the other. Item discriminability refers to how effectively a particular item differentiates individuals based on their position on the underlying IBP scale (eg, higher vs lower latent IBP levels). In non-uniform DIF, the difference in item functioning between subgroups varies across the latent IBP scale, depending on the individual’s IBP level.

Additionally, after conducting DIF testing for each item, we evaluated the equality of all non-DIF items collectively. If this equality was supported (eg, nonsignificant difference), we proceeded to sequentially test the equality of the properties of latent variance and latent mean for the IBP construct to finalise the IRT model by assessing whether the properties the latent construct are the same between the groups or not.

## RESULTS

### Unidimensionality in CFA of IBP criteria

There were 1511 patients with ARQ IBP questionnaires analysed. Among the IBP criteria items, the proportion of missing data ranged from 3 to 821, with the larger ones occurring on ‘adult onset <40’ (821), ‘adult onset <45’ (751), ‘adult onset <30’ (531), and ‘insidious onset’ (381), and the smaller ones on ‘spinal pain’ (3), ‘lower back pain’ (3), ‘morning stiffness’ (101), ‘improvement with exercise’ (171), ‘pain in the 2^nd^ half of the night’ (175), ‘alternating buttock pain’ (91), and ‘improvement with exercise but not rest’ (229). After imputing those missing data with the missForest algorithm, for all IBP criteria (eg, Calin, ESSG, Berlin 8a and Berlin 7b), CFA favoured 1-factor models with nonsignificant chi-squared test, improved RMSEA, observed as compared to 2-factor models (Table 2). This suggests that the criteria questionnaires do test one underlying conditions, (eg, IBP), as opposed to testing different conditions of back pain.

### Age-based differential item functioning observed in all IBP criteria

DIF analysis for age groups revealed DIF in at least 1 item for all IBP criteria, suggesting systemic difference between younger and older patients’ responses (Table 3). The Berlin 7b’s ‘adult onset <30 years old’ item displayed non-uniform DIF, indicating the relationship between the latent IBP and the item varied between the 2 age groups. Specifically, even when the latent IBP level was the same between the 2 groups, the item was more likely to be endorsed by younger patients at lower latent IBP levels but more likely to be endorsed by older patients at higher IBP levels. Non-uniform DIF/systematic bias for the items ‘adult onset <40/<45’ and ‘morning stiffness’ were observed in Calin and ESSG criteria. respectively. Other items in the Berlin 7b, Calin, and ESSG criteria did not show DIF for age groups. Collective equality testing of non-DIF items did not hold for either Berlin 7b or Calin, but for ESSG. For ESSG, the variance of the latent IBP was the same between the groups, but the mean of the latent IBP was lower for the older group than the younger one.

In contrast, the Berlin 8a IBP criteria showed only one uniform DIF item, indicating that older individuals found it harder to answer ‘yes’ to the ‘improvement with exercise/no improvement with rest’ item when their latent IBP level is the same as the younger ones but still had similar IBP discriminative ability (the ability to identify the IBP compared to non-IBP) unlike the other IBP criteria. For Berlin 8a, a model with simultaneous constraints for all the identified non-DIFs items (partial invariance model) was as good as the reference model with only the anchor variable constrained. Further, the variance and mean of the latent IBP variable were equal between the age groups. This does suggest that the Berlin 8a may be equally applicable to both older and younger patients with adjustment or modification for the ‘improvement with exercise/no improvement with rest’ item.

### Race and gender analyses show minimal DIF in IBP criteria

In our race and gender analyses, DIF was only found in the Berlin 8a lower back pain item for gender groups (Supplementary Tables S1 and S2). None of the other IBP criteria had DIF observed. For race, all the equality tests held indicating that the mean and variance of the latent IBP was the same between the 2 groups in all the IBP criteria, except the mean of the latent IBP in Berlin7b. The mean of the latent IBP was higher for the non-white group than the white group. Thus, across race groups we observed similar responses at both an individual item/question level as well as the IBP criteria collectively except for the Berlin 7b.

In contrast, collective equality testing of non-DIF items held only for Berlin 7b in men compared with women. The mean and variance of the latent IBP were also equal between the 2 groups (Supplementary Table S1). Thus, individual IBP questions do not systematically favour one gender. However, at an overall scale level, there still may be meaningful differences in how IBP criteria operate for men compared to women despite their similar item function.

### Sensitivity analyses confirm similar DIF patterns

To ensure the robustness of our findings, we conducted sensitivity analyses using complete cases and applied the same constrained baseline approach for anchor item identification. The results were largely consistent with those from the imputed dataset. For example, age-related DIF in the Berlin 8a item ‘improvement with exercise but not with rest’ remained evident. Overall, age-based comparisons continued to show the most prominent DIF patterns (see Supplementary Table S3).

In contrast, race and gender comparisons revealed minimal DIF in the complete-case analysis, which is consistent with our main findings. Specifically, no DIF was observed across any of the IBP criteria in the gender-based analysis (Supplementary Table S4). However, in the complete case race-based analysis, the Calin and ESSG criteria did exhibit evidence of DIF, suggesting a potential need for cautious interpretation of these criteria in racially diverse populations (Supplementary Table S5).

## DISCUSSION

This study supports the use of a single underlying construct in IBP criteria, which aligns with the unidimensional nature of IBP assessment. In contrast, when studying subgroups on DIF and equality testing, age and gender, respectively, introduce significant variability. This could impact the epidemiologic research and clinical utility of these criteria. The identified DIF items indicate potential biases that should be addressed to ensure accurate recognition of IBP. Particularly, the non-uniform DIF found in all of the tested IBP criteria for older patients with the exception of the Berlin 8a criteria suggests issues using these constructs in older patient populations. Although individual items in our gender analyses generally did not show DIF, suggesting these criteria sets may be misleading in identification of IBP.

The results add to our understanding of IBP. To our knowledge, this study is the first to examine structural validity of the IBP criteria and test their psychometric performance in different age, ethnic, and gender contexts. This shows that the current criteria may benefit from refinement to reduce bias and improve accuracy when studying SpA subgroups such as older adults and across genders. It also shows that perhaps instead of a dichotomous understanding of the presence or nonpresence of IBP, there instead needs to be a more continuous approach to identification of IBP, with a certain threshold that differs based on the underlying subpopulation studied. For example, older patients may require more elements or down-weighting of items that are more ubiquitous in this population, perhaps unrelated to the IBP construct. The non-equality observed between men and women also reflects how SpA can manifest differently across genders as previously published literature suggests [19–24].

Our study had limitations. The more widely applied ASAS IBP criteria were not available/tested in this dataset given the timeline of the ARQ development and NHANES 2009–2010 dataset. This requires further, independent analyses, although similar individual IBP questions/items are a part of the ASAS criteria. Our study does not include direct SpA diagnosis data, limiting the ability to determine the predictive value of IBP criteria for SpA; the presence or lack thereof of SpA at an individual level was not available in the NHANES 2009–2010 reporting [25]. Thus, the positive and negative predictive values of IBP positivity/negativity for the presence of SpA cannot be calculated in this study. Missing IBP items among the NHANES 2009–2010 cohort could also affect the results and interpretability. Future studies should incorporate SpA-confirmed cohorts to validate these findings and assess their clinical applicability.

In a clinical context, IBP requires clinical assessment dependent on skill and experience of the examiner. Therefore, we feel that IBP criteria should not be applied as the sole determinant of the presence of IBP for clinical trial enrollment. We would suggest currently that expert, gestalt, clinical judgement remains the gold standard for determining IBP. Improper use of these criteria sets may lead to over or underdiagnosis in individual patients as these items resonate differently with them. This has implications as newer research tools including natural language processing and artificial intelligence that find electronic health record text and other identifiers suggest of IBP can be compared to criteria performance in different patient subgroups/contexts. This may help further tool development for epidemiological studies in SpA.

This study underscores the importance of understanding the dimensionality and differential functioning of IBP criteria. Although the criteria effectively capture IBP features, demographic differences necessitate further investigation and potential adjustments to ensure proper recognition. Specifically, our findings highlight the need for tailored, clinical assessment of IBP with physicians cognizant of potential biases introduced by age, race, and gender. Given the observed demographic variability, clinicians should interpret IBP criteria cautiously, particularly in older adults and women, where differential performance was noted. These findings suggest that IBP screening tools may require tailored adjustments to improve diagnostic accuracy across populations. Older patients are more likely to respond ‘yes’ to questions about chronic lower back pain and have more difficulty noting improvement with exercise/worsening with rest compared to younger patients. Women have greater variability in their IBP condition in these criterion sets. This may reflect how IBP in women is poorly reflected in the established criterion sets.

Further research should further delve into more diverse populations to uncover potential additional demographics factors influencing IBP criteria performance. This will allow for better detection of the presence of IBP and has implications for educating patients and healthcare providers in IBP symptom nuances in different patient population contexts. This will lead to more accurate IBP recognition prompting earlier axSpA evaluation. Earlier axSpA evaluation can reduce diagnostic delay, leading to earlier treatments and potentially enhancing patient outcomes.

## Supplementary Material

Supplemental Tables 1-5

Supplementary materials

Supplementary material associated with this article can be found in the online version at doi:10.1016/j.ero.2025.04.006.

## Figures and Tables

**Table 1:** Study sample for the NHANES 2009–2010 US inflammatory back pain survey [6]^a^

Selected characteristics	N	%

Total study sample	5103	100.00
Age groups (y)		
20–35	1649	32.3
30–49	1539	30.2
50–69	1915	37.5
Gender		
Males	2472	48.4
Females	2631	51.6
Race/ethnicity subgroups		
Mexican-Americans	1024	20.1
Other Hispanics	576	11.3
Non-Hispanic Whites	2244	44.0
Non-Hispanic Blacks	963	18.9
All others	296	5.8
Total axial pain sample^b^	980	100.0
Current pain	873	89.1
No current pain	107	10.9
Age-at-onset of pain (y)		
<20	164	16.8
20–29	247	25.3
30–44	306	31.4
≥45	258	26.5
Duration of pain (y)		
<1	50	5.2
1–2	154	16.0
3–10	343	35.7
>10	415	43.1
Temporal pattern of pain		
Constant, never goes away	660	67.5
Episodic, no relief >1 mo	178	18.2
Episodic, pain relief >1 mo	123	12.6
One pain episode only	17	1.7

NHANES, National Health and Nutrition Examination Survey.

Subgroup totals for pain age at onset, duration, and temporal pattern are reduced by item non-response.

a Adapted from Weisman et al [6].

b Axial pain variable = survey participants with a prior episode of neck, upper, mid or low back, or buttocks pain for 3 months or more.

**Table 2:** Confirmatory factor analysis of NHANES 2009–2010 inflammatory back pain criteria

Criteria	Model	Chi-squared	CFI	RMSEA	SRMR

Calin	1-factor	5.614	0.984	0.005	0.034
	2-factor	4.490	0.933	0.016	0.029
	Difference	1.124, *p* > .05			
ESSG	1-factor	15.115	0.889	0.020	0.034
	2-factor	15.108	0.856	0.036	0.034
	Difference	0.007, *p* > .05			
Berlin 7b	1-factor	2.531	0.965	0.007	0.025
	2-factor	NA^a^	NA	NA	NA
	Difference	NA			
Berlin 8a	1-factor	6.144	0.996	0.007	0.026
	2-factor	4.736	0.989	0.016	0.024
	Difference	1.408, *p* > .05			

CFI, comparative fit index; ESSG, European Spondyloarthropathy Study Group; NA, not applicable; NHANES, National Health and Nutrition Examination Survey; RMSEA, root mean square error of approximation; SRMR, standardised root mean squared residual.

a NA = not applicable due to the inability to achieve convergence for the 2-factor model.

**Table 3:** DIF age analysis of younger (20–49) vs (50)+ older NHANES 2009–2010 patients(n = 1511)

Variable tested	Chi-square diff	*P* value

Berlin 8a^a^		
DIF testing for individual items		
Second half of the night awakening due to back pain (anchor variable)	-	-
Lower back pain	4.126	.127
Alternating buttock pain	7.535	.023
Morning stiffness	2.959	.228
**Improvement with exercise but not with rest**	**15.778**	**<.001**
Improvement with exercise but not with rest slope only	1.045	.307
**Improvement with exercise but not with rest intercept only**	**12.786**	**<.001**
All non-DIF item equality testing		
Lower back pain, alternating buttock pain, morning stiffness, slope of improvement with exercise but not with rest equality	15.43	.031
Latent variable equality testing		
Factor variance equality	2.978	.084
Factor mean equality	0.448	.503
Berlin 7b^b^		
DIF testing for individual items		
Morning stiffness (anchor variable)	-	-
Lower back pain	4.809	.09
Improvement with exercise but not with rest	6.127	.047
**Age of onset <30 y**	**46.302**	**<.001**
**Age of onset <30 y slope only**	**15.196**	**<.001**
Age of onset <30 y intercept only	3.284	.07
All non-DIF item equality testing^c^		
**Lower back pain, improvement with exercise but not with rest, intercept of age of onset < 30 y equality**	**42.953**	**<.001**
ESSG^a^		
DIF testing for individual items		
Improvement with exercise (anchor variable)	-	-
**Age onset <45 y**	**15.316**	**<.001**
Age onset <45 y slope only	2.947	.086
Age onset <45 y intercept only	4.045	.044
Insidious onset	8.167	.017
**Morning stiffness**	**12.426**	**.002**
**Morning stiffness slope only**	**5.743**	**.017**
Morning stiffness intercept only	0.09	.764
Spinal pain	8.01	.018
All non-DIF item equality testing		
all items equality	15.43	.031
Latent variable equality testing		
Factor variance equality	0.067	.795
**Factor mean equality**	**644.733**	**<.001**
Calin^a^		
DIF testing for individual items		
Improvement with exercise (anchor variable)	-	-
**Age onset <40 y**^a^	**11.746**	**.003**
**Age onset <40 y slope only**^a^	**13.306**	**<.001**
Age onset <40 y intercept only	2.279	.131
Insidious onset	7.815	.02
**Morning stiffness**^a^	**14.563**	**.001**
**Morning stiffness slope only**^a^	**264.725**	**<.001**
**Morning stiffness intercept only**^a^	**270.377**	**<.001**
Spinal pain	8.034	.018
All non-DIF item equality testing^c^		
Insidious onset, spinal pain, intercept of age onset <40 y equality	**39.558**	**<.001**

DIF, differential item functioning; ESSG, European Spondyloarthropathy Study Group; NHANES, National Health and Nutrition Examination Survey.

Boldface text and values are items below preselected p-value signficance threshold.

a *p* value significance threshold of 0.0125 (.05/4) for Bonferroni adjustment applied.

b *p* value significance threshold of 0.017 (.05/3) for Bonferroni adjustment applied.

c Latent variable property testing not performed due to all non-DIF items equality testing not supported.

## What is already known on this topic

- Inflammatory back pain (IBP) criteria applied at a population level have varying prevalence and varying relationship to spondyloarthritis (SpA) depending on the criteria and population studied.

### WHAT THIS STUDY ADDS

- IBP criteria may have a single, underlying construct. Psychometric testing of different subpopulations however, particularly based on age and gender, have significant varying responses to these IBP criteria that lead to questions regarding their validity at a population level.

### HOW THIS STUDY MIGHT AFFECT RESEARCH, PRACTICE OR POLICY

- These findings suggest that modification and/or weighting needs to be applied to IBP criteria depending on the subpopulation. Furthermore, it reinforces that IBP determination cannot be made applying criteria; assessment by clinicians experienced in SpA patient care remains the best tool for the detections of IBP at a patient-level.
