# Supplementary material for: Psychometrics of inflammatory back pain criteria in the US population
Source: EULAR Rheumatol Open. Author manuscript; Available in PMC 2026 Jan 8. (PMC12777957; doi:10.1016/j.ero.2025.04.006)
Supplement: Supplemental Tables 1-5 [file NIHMS2127350-supplement-Supplemental_Tables_1-5.docx]

| Supplemental Table 1. DIF Race Analysis (White vs. Non-White) NHANES 2009-2010 patients (n=1511) | | |
| --- | --- | --- |
| Variable tested | Chi-square Diff | P value |
| Berlin 8a* |  |  |
| DIF testing for individual items |  |  |
| Alternating buttock pain (anchor variable) | - | - |
| Lower back pain | 0.427 | 0.808 |
| Second half of the night awakening due to back pain | 2.49 | 0.288 |
| Morning stiffness | 0.316 | 0.854 |
| Improvement with exercise but not with rest | 1.651 | 0.438 |
| All non-DIF item equality testing |  |  |
| All items equality | 10.879 | 0.209 |
| Latent Variable equality testing |  |  |
| Factor variance equality | 0.574 | 0.449 |
| **Factor mean equality** | **11.158** | **0.001** |
| Berlin 7b† |  |  |
| DIF testing for individual items |  |  |
| Lower back pain (anchor variable) | - | - |
| Age of onset <30 years | 3.301 | 0.192 |
| Morning stiffness | 0.203 | 0.903 |
| Improvement with exercise but not with rest | 0.005 | 0.998 |
| All non-DIF item equality testing |  |  |
| All items equality | 3.496 | 0.744 |
| Latent Variable equality testing |  |  |
| Factor variance equality | 0.635 | 0.425 |
| Factor mean equality | 0.003 | 0.959 |
| ESSG* |  |  |
| DIF testing for individual items |  | |
| Spinal Pain (anchor variable) | - | - |
| Improvement with exercise | 2.921 | 0.232 |
| Age onset <45 years | 0.664 | 0.717 |
| Insidious onset | 3.275 | 0.194 |
| Morning stiffness | 0.84 | 0.84 |
| All non-DIF item equality testing |  |  |
| all items equality | 8.177 | 0.416 |
| Latent Variable equality testing |  |  |
| Factor variance equality | 0.455 | 0.5 |
| Factor mean equality | 0.854 | 0.355 |
| Calin* |  |  |
| DIF testing for individual items |  | |
| Spinal Pain (anchor variable) | Anchor variable | |
| Age onset <40 years | 3.84 | 0.147 |
| Insidious onset | 2.522 | 0.283 |
| Morning stiffness | 1.547 | 0.461 |
| Improvement with exercise | 3.115 | 0.211 |
| All non-DIF item equality testing |  |  |
| All items equality | 9.625 | 0.292 |
| Latent Variable equality testing |  |  |
| Factor variance equality | 0.487 | 0.485 |
| Factor mean equality | 0.953 | 0.329 |
| ***** p-value significance threshold of 0.0125 (.05/4) for Bonferonni adjustment applied  † p-value significance threshold of 0.017 (.05/3) for Bonferonni adjustment applied | | |

| Supplemental Table 2. DIF Gender Analysis (Men vs. Women) NHANES 2009-2010 patients (n=1511) | | |
| --- | --- | --- |
| Variable tested | Chi-square Diff | P value |
| Berlin 8a* |  |  |
| DIF testing for individual items |  | |
| Second half of the night awakening due to back pain | Anchor variable | |
| **Lower back pain** | **8.813** | **0.012** |
| Lower back pain slope only | 3.078 | 0.079 |
| Lower back pain intercept only | 2.306 | 0.129 |
| Alternating buttock pain | 5.312 | 0.07 |
| Morning stiffness | 0.846 | 0.655 |
| Improvement with exercise but not with rest | 4.864 | 0.088 |
| All non-DIF item equality testing**†** |  |  |
| **All items equality** | **22.637** | **0.004** |
| Berlin 7b‡ |  |  |
| DIF testing for individual items |  | |
| Lower back pain | Anchor variable | |
| Age of onset <30 years | 0.799 | 0.671 |
| Morning stiffness | 4.448 | 0.108 |
| Improvement with exercise but not with rest | 0.438 | 0.803 |
| All non-DIF item equality testing**†** |  |  |
| All items equality | 7.315 | 0.293 |
| Latent Variable equality testing |  |  |
| Factor variance equality | 0.79 | 0.374 |
| Factor mean equality | 0.201 | 0.654 |
| ESSG* |  |  |
| DIF testing for individual items |  | |
| Spinal Pain | Anchor variable | |
| Improvement with exercise | 6.267 | 0.044 |
| Age onset <45 years | 1.522 | 0.467 |
| Insidious onset | 8.292 | 0.016 |
| Morning stiffness | 7.864 | 0.049 |
| All non-DIF item equality testing**†** |  |  |
| **all items equality†** | **32.096** | **<0.001** |
| Calin* |  |  |
| DIF testing for individual items |  | |
| Spinal Pain | Anchor variable | |
| Age onset <40 years | 1.713 | 0.425 |
| Insidious onset | 8.593 | 0.014 |
| Morning stiffness | 3.083 | 0.214 |
| Improvement with exercise | 8.387 | 0.015 |
| All non-DIF item equality testing**†** |  |  |
| **All items equality** | **28.091** | **<0.001** |
| * p-value significance threshold of 0.0125 (.05/4) for Bonferonni adjustment applied  † latent variable property testing not performed due to all- non-DIF items equality testing not supported  ‡ p-value significance threshold of 0.017 (.05/3) for Bonferonni adjustment applied | | |

| Supplemental Table 3. DIF Age analysis of Younger (20-49) vs. (50)+ Older NHANES 2009-2010 patients complete cases* | | |
| --- | --- | --- |
| Variable tested | Chi-square Diff | P value |
| Berlin 8a† |  |  |
| DIF testing for individual items |  |  |
| Second half of the night awakening due to back pain (anchor variable) | - | - |
| Lower back pain | **5.155** | **0.076** |
| Alternating buttock pain | 5.848 | 0.054 |
| Morning stiffness | 2.034 | 0.362 |
| **Improvement with exercise but not with rest** | 9.051 | 0.011 |
| Berlin 7b‡ |  |  |
| DIF testing for individual items |  |  |
| Morning stiffness (anchor variable) | - | - |
| **Lower back pain** | **10.163** | **0.006** |
| Improvement with exercise but not with rest | 5.568 | 0.062 |
| **Age of onset <30 years** | **16.983** | **<0.001** |
| ESSG† |  |  |
| DIF testing for individual items |  |  |
| Improvement with exercise (anchor variable) | - | - |
| **Age onset <45 years** | **20.124** | **<0.001** |
| Insidious onset | 8.167 | 0.017 |
| Morning stiffness | 5.462 | 0.065 |
| **Spinal Pain** | **31.245** | **<0.001** |
| Calin† |  |  |
| DIF testing for individual items |  |  |
| Improvement with exercise (anchor variable) | - | - |
| **Age onset <40 years** | **23.837** | **<0.001** |
| **Insidious onset** | **13.568** | **0.001** |
| Morning stiffness | 2.372 | 0.305 |
| Spinal Pain | 0.328 | 0.849 |
| * n=1198, 884, 642, 641 for Berlin 8a, Berlin 7b, ESSG and Calin complete cases, respectively.  † p-value significance threshold of 0.0125 (.05/4) for Bonferonni adjustment applied  ‡ p-value significance threshold of 0.017 (.05/3) for Bonferonni adjustment applied | | |

| Supplemental Table 4. DIF Gender Analysis (Men vs. Women) NHANES 2009-2010 patient complete cases* | | |
| --- | --- | --- |
| Variable tested | Chi-square Diff | P value |
| Berlin 8a† |  |  |
| DIF testing for individual items |  | |
| Second half of the night awakening due to back pain | Anchor variable | |
| Lower back pain | 6.101 | 0.047 |
| Alternating buttock pain | 8.398 | 0.015 |
| Morning stiffness | 0.313 | 0.855 |
| Improvement with exercise but not with rest | 0.447 | 0.8 |
| Berlin 7b‡ |  |  |
| DIF testing for individual items |  | |
| Lower back pain | Anchor variable | |
| Age of onset <30 years | 1.101 | 0.577 |
| Morning stiffness | 1.27 | 0.53 |
| Improvement with exercise but not with rest | 0.003 | 0.999 |
| ESSG† |  |  |
| DIF testing for individual items |  | |
| Spinal Pain | Anchor variable | |
| Improvement with exercise | 3.661 | 0.16 |
| Age onset <45 years | 2.442 | 0.295 |
| Insidious onset | 6.307 | 0.043 |
| Morning stiffness | 0.796 | 0.85 |
| Calin† |  |  |
| DIF testing for individual items |  | |
| Spinal Pain | Anchor variable | |
| Age onset <40 years | 1.534 | 0.464 |
| Insidious onset | 5.757 | 0.056 |
| Morning stiffness | 1.37 | 0.504 |
| Improvement with exercise | 3.538 | 0.17 |
| * n=1198, 884, 642, 641 for Berlin 8a, Berlin 7b, ESSG and Calin complete cases, respectively.  † p-value significance threshold of 0.0125 (.05/4) for Bonferonni adjustment applied  ‡ p-value significance threshold of 0.017 (.05/3) for Bonferonni adjustment applied | | |

| Supplemental Table 5. DIF Race Analysis (White vs. Non-White) NHANES 2009-2010 patients complete cases* | | |
| --- | --- | --- |
| Variable tested | Chi-square Diff | P value |
| Berlin 8a† |  |  |
| DIF testing for individual items |  |  |
| Alternating buttock pain (anchor variable) | - | - |
| Lower back pain | 1.152 | 0.562 |
| Second half of the night awakening due to back pain | 3.159 | 0.206 |
| Morning stiffness | 0.687 | 0.709 |
| Improvement with exercise but not with rest | 5.919 | 0.052 |
| Berlin 7b‡ |  |  |
| DIF testing for individual items |  |  |
| Lower back pain (anchor variable) | - | - |
| Age of onset <30 years | 6.223 | 0.045 |
| Morning stiffness | 0.608 | 0.738 |
| Improvement with exercise but not with rest | 0.9 | 0.638 |
| ESSG† |  |  |
| DIF testing for individual items |  | |
| Spinal Pain (anchor variable) | - | - |
| Improvement with exercise | 0.547 | 0.761 |
| **Age onset <45 years** | **9.444** | **0.009** |
| Insidious onset | 3.214 | 0.2 |
| Morning stiffness | 3.873 | 0.275 |
| Calin† |  |  |
| DIF testing for individual items |  | |
| Spinal Pain (anchor variable) | Anchor variable | |
| **Age onset <40 years** | **12.633** | **0.002** |
| **Insidious onset** | **14.167** | **0.001** |
| Morning stiffness | 1.159 | 0.56 |
| Improvement with exercise | 0.066 | 0.967 |

^*^n=1198, 884, 642, 641 for Berlin 8a, Berlin 7b, ESSG and Calin complete cases, respectively.

^†^p-value significance threshold of 0.0125 (.05/4) for Bonferonni adjustment applied

^‡^p-value significance threshold of 0.017 (.05/3) for Bonferonni adjustment applied
